# Identification of genomic regions associated with soybean responses to off-target dicamba exposure

**DOI:** 10.3389/fpls.2022.1090072

**Published:** 2022-12-09

**Authors:** Caio Canella Vieira, Diego Jarquin, Emanuel Ferrari do Nascimento, Dongho Lee, Jing Zhou, Scotty Smothers, Jianfeng Zhou, Brian Diers, Dean E. Riechers, Dong Xu, Grover Shannon, Pengyin Chen, Henry T. Nguyen

**Affiliations:** ^1^ Fisher Delta Research, Extension, and Education Center, Division of Plant Science and Technology, University of Missouri, Portageville, MO, United States; ^2^ Agronomy Department, University of Florida, Gainesville, FL, United States; ^3^ Biological Systems Engineering, University of Wisconsin-Madison, Madison, WI, United States; ^4^ Division of Plant Science and Technology, University of Missouri, Columbia, MO, United States; ^5^ Department of Crop Sciences, University of Illinois, Urbana, IL, United States; ^6^ Department of Electrical Engineering and Computer Science, Bond Life Sciences Center, University of Missouri, Columbia, MO, United States

**Keywords:** soybean, plant breeding, dicamba, synthetic auxin, genomics, GWAS, G×E

## Abstract

The widespread adoption of genetically modified (GM) dicamba-tolerant (DT) soybean was followed by numerous reports of off-target dicamba damage and yield losses across most soybean-producing states. In this study, a subset of the USDA Soybean Germplasm Collection consisting of 382 genetically diverse soybean accessions originating from 15 countries was used to identify genomic regions associated with soybean response to off-target dicamba exposure. Accessions were genotyped with the SoySNP50K BeadChip and visually screened for damage in environments with prolonged exposure to off-target dicamba. Two models were implemented to detect significant marker-trait associations: the Bayesian-information and Linkage-disequilibrium Iteratively Nested Keyway (BLINK) and a model that allows the inclusion of population structure in interaction with the environment (G×E) to account for variable patterns of genotype responses in different environments. Most accessions (84%) showed a moderate response, either moderately tolerant or moderately susceptible, with approximately 8% showing tolerance and susceptibility. No differences in off-target dicamba damage were observed across maturity groups and centers of origin. Both models identified significant associations in regions of chromosomes 10 and 19. The BLINK model identified additional significant marker-trait associations on chromosomes 11, 14, and 18, while the G×E model identified another significant marker-trait association on chromosome 15. The significant SNPs identified by both models are located within candidate genes possessing annotated functions involving different phases of herbicide detoxification in plants. These results entertain the possibility of developing non-GM soybean cultivars with improved tolerance to off-target dicamba exposure and potentially other synthetic auxin herbicides. Identification of genetic sources of tolerance and genomic regions conferring higher tolerance to off-target dicamba may sustain and improve the production of other non-DT herbicide soybean production systems, including the growing niche markets of organic and conventional soybean.

## Introduction

Soybean [*Glycine max* (L.) Merr.] plays a multifaceted role in the global agricultural trade, economy, and food security due to its unique seed composition (Gale et al., 2019; Liu et al., 2020; Vieira and Chen, 2021). As a major source of protein and vegetable oil, soybean is widely used in the food, feed, and biofuel industries (Hartman et al., 2011; Vogel et al., 2021). In the United States, approximately 95% of the soybean acreage is grown with genetically-engineered herbicide-tolerant cultivars, of which nearly 55% are grown using the dicamba-tolerance trait (DT, 3,6-dichloro-2-methoxybenzoic acid) (Bayer CropScience LLC, 2021; USDA Economic Research Service, 2022). Dicamba is a synthetic auxin (Group 4 herbicide) that triggers rapid and uncontrolled growth of the stems, petioles, and leaves, often leading to plant death in sensitive dicots (Environmental Protection Agency, 2006; Grossmann, 2010). A distinguished characteristic of Group 4 herbicides is their high vapor pressure. Dicamba in specific has a vapor pressure of 2.0×10^-5^ mm HG at 25 C, which significantly increases the occurrence of off-target movement to adjacent fields (Behrens and Lueschen, 1979; Egan and Mortensen, 2012; Werle et al., 2018; Wechsler et al., 2019; Chism et al., 2020; Wagman et al., 2020). By comparison, glyphosate (*N*-(phosphonomethyl)glycine) has high water solubility and vapor pressure of 1.9×10^−7^ mm HG at 25°C (Battaglin et al., 2005).

The widespread adoption of DT cropping systems led to numerous cases of off-target damage to non-DT soybean as well as several other dicots plant species (Bradley, 2017; Bradley, 2018; Wechsler et al., 2019; Chism et al., 2020; Wagman et al., 2020). Between 2017 and 2021, the Environmental Protection Agency (EPA) received over 10,500 reports of dicamba-related injuries in various non-DT vegetations in 29 of the 34 states where the use of dicamba on DT crops is authorized (Echeverria, 2020; Tindall et al., 2021). Soybean is naturally sensitive to dicamba, and symptoms include crinkling and cupping of immature leaves, epinasty, plant height reduction, chlorosis, death of apical meristem, malformed pods, and ultimately yield reduction (Weidenhamer et al., 1989; Andersen et al., 2004; Grossmann, 2010; Kniss, 2018; Canella Vieira et al., 2022b). The severity of the symptoms and yield loss differ based on the timing of exposure (growth stage), dosage, frequency, and duration of exposure. It is well known that the expression of a phenotype is a function of the genotype (G), the environment (E), and the differential phenotypic response of genotypes to different environments (G×E) (de Leon et al., 2016). However, information is lacking regarding the effect of different genetic backgrounds and identification of genomic regions affecting the severity of symptoms and yield loss caused by off-target dicamba in soybean.

With the advances in the availability of high-dimensional genomic data (Song et al., 2013; Song et al., 2020) and comprehensive statistical models (Zhang et al., 2010; Liu et al., 2016; Huang et al., 2019), genome-wide association studies (GWAS) have been largely used as a common approach to help reveal the underlying genetic basis of a trait of interest during the past decade. With several thousand to millions of single nucleotide polymorphisms (SNPs), GWAS captures significant associations between the trait of interest and molecular markers using a broad range of linear or logistic regression models as well as machine and deep learning algorithms. In soybean, GWAS has unveiled the genetic architecture of multiple economic-important traits, including tolerance to biotic (Vuong et al., 2015; Chang et al., 2016; Canella Vieira et al., 2022c) and abiotic (Valliyodan et al., 2016; Zeng et al., 2017; Wu et al., 2020) stressors, seed composition (Hwang et al., 2014; Bandillo et al., 2015), agronomic (Sonah et al., 2015; Zhang et al., 2015), physiology-efficient (Lopez et al., 2022), as well as yield (Yoosefzadeh-Najafabadi et al., 2021) and domestication-related traits (Han et al., 2016; Wang et al., 2016b). To date, GWAS has not been conducted to identify genomic regions associated with soybean tolerance to dicamba or other herbicides. Therefore, the objective of this study was to identify significant marker-trait associations regulating the response of genetically diverse soybean accessions to off-target dicamba grown under prolonged exposure to dicamba under field conditions. Natural tolerance to off-target dicamba may sustain and improve the production of other non-DT herbicide soybean production systems, including the growing niche markets of organic and conventional soybean.

## Materials and methods

### Plant materials and data collection

A total of 382 genetically diverse soybean accessions with maturity groups (MG) ranging from MG 3 to 5 were used in this study. These comprise a subset of the USDA Soybean Germplasm Collection and originated from 15 countries, including Algeria (2), China (226), Costa Rica (1), Georgia (2), Indonesia (1), Japan (38), Nepal (1), North Korea (20), Russia (5), South Africa (1), South Korea (32), Taiwan (3), United States (40), and Vietnam (5). Five accessions have unknown origins. The USDA Soybean Germplasm Collection was genotyped with the SoySNP50K BeadChip (Song et al., 2013) and the data has been made available by the authors (Song et al., 2015) at SoyBase Genetics and Genomics Database (http://soybase.org/snps/download.php). SNPs were converted to numerical format (0, 1, and 2 for the homozygous minor allele, heterozygous, and homozygous major allele, respectively), and were excluded based on minor allele frequency (MAF)< 0.05 resulting in 31,957 SNPs. The across-genome SNP density on a chromosome basis was 1,598, ranging from 1,186 (Chr. 11) to 2,619 (Chr. 18).

Field trials were conducted in three environments for two years (2020-2021) using a two-replicate randomized complete block design at the Lee Farm in Portageville, MO (36°23’44.2”N lat; 89°36’52.3”W long) and Rhodes Farm in Clarkton, MO (36°29′14.8″N lat; 89°57′39.0″W long). Each plot consisted of a single 2.13 m long row spaced 0.76 m apart. The Lee Farm and Rhodes Farm have been exposed to prolonged and homogeneously distributed off-target dicamba damage since 2017, where significant yield losses due to off-target dicamba exposure have been reported between non-DT and DT soybean genotypes (Canella Vieira et al., 2022a; Chen et al., 2022; Canella Vieira et al, 2022b). Off-target dicamba exposure was a result of dicamba volatilization from nearby cropping systems consisting of DT soybean and cotton.

Each year, genotypes were visually assessed for off-target dicamba damage once in the early reproductive stage between R1 to R3 (approximately 100 to 130 DAP) (Fehr et al., 1971). Lines were rated on a 1 to 5 scale with 0.5 increments following the criteria described by Canella Vieira et al. (2022b). In summary, a rating of 1 showed none to minimal visual dicamba damage symptoms, including the typical crinkling and cupping of the newly-developing leaves, reduced canopy coverage, and plant stunting; a rating of 2 showed moderate tolerance with limited cupping of the newly-developing leaves and no visual impact on canopy coverage and vegetative growth; a 3 rating showed accentuated cupping of the newly-developing leaves and moderate reduction in canopy area and vegetative growth; a 4 rating showed severe cupping of the newly-developing leaves and pronounced reduction in canopy area and vegetative growth, and a rating of 5 showed extreme dicamba damage symptoms including severe cupping of the newly-developing leaves and intense reduction in canopy coverage and vegetative growth.

Adjusted means across environments were calculated using the function ‘*ls_means*’ of the R (R Core Team, 2022) package ‘*lmerTest*’ (Kuznetsova et al., 2017) based on a mixed-effects linear model conducted with the package ‘*lme4’* (Bates et al., 2015). The model included the fixed effect of genotype, the random interaction between genotype and environment (G×E), the random effect of environment, and the nested random effect of replication within the environment. To measure the consistency and inter-relatedness of the damage ratings across environments, Cronbach’s alpha (α) score (Cronbach, 1951) was calculated each year using the R package ‘*psych*’ (Revelle, 2021) based on Eq. 1.


Eq. 1 $$\alpha =\;\frac{k\;\times \;\bar{c}}{\bar{v}+\left(k-1\right)\bar{c}}$$

Where *k* represents the number of observations of off-target dicamba damage; $\bar{c}$ is the average inter-item covariance of off-target dicamba damage scores between each pair of environments averaged for all pairs of environments; and $\bar{v}$ is the average variance of off-target dicamba damage scores across all environments.

### Genome-wide association study

Two models have been implemented to detect significant marker-trait associations. The Bayesian-information and Linkage-disequilibrium Iteratively Nested Keyway (BLINK) model (Huang et al., 2019) was conducted using the adjusted means across environments as phenotypic input in the R package ‘*GAPIT*’ (Lipka et al., 2012). It is an enhanced methodology based on the Fixed and Random Model Circulating Probability Unification (FarmCPU) (Liu et al., 2016). In summary, FarmCPU conducts two fixed-effect models iteratively and a filtering process to select a set of pseudo-SNPs that are not in linkage disequilibrium with each other as covariates. The first model tests one SNP at a time with multiple associated markers fitted as covariates to account for population stratification. The main goal is to control false positives and reduce false negatives, as well as calculate the *p*-values for all testing SNPs. The second model selects the covariate markers to directly control false associations instead of kinship. BLINK eliminates the requirement that genes underlying a trait are equally distributed across the genome, and also replaces the Restricted Maximum Likelihood (REML) with Bayesian Information Content (BIC) in a fixed-effect model to boost computing speed. The detailed methodology can be found in Huang et al. (2019).

To account for the variable patterns of genotype responses to off-target dicamba in different environments, a model that allows the inclusion of the population structure in interaction with environments was considered. The model was fitted with ASREML‐R (VSN‐International, England). Considering that *y*_*ijk*_ represents the kth (k=1,2) response of the ith (i=1,2,…,382) genotype in the jth (j=1,…,3) environment, the GWAS was conducted using the following linear mixed model in matrix form:


Eq. 2 $$y=E+R:E+PC_{1:10}+E\times PC_{1}+\;E\times PC_{2}+\ldots +E\times PC_{10}+x_{k}+L+e$$

Where ***E*** corresponds to the main effect of the environments; ***R:E*** represents the effect of the replicates nested within environments; *PC*_1:10_ are the first 10 principal components (PC) derived from decomposing the **G** matrix using a principal component analysis (PCA) and are included in the model for correcting for population structure; the interactions between the first 10 components *PC*_1:10_ and environments were also included with the  *E*×*PC*_*j*_ term; the *x*_*k*_ term corresponds to the *k* ^th^ molecular marker associated with *β*_*k*_ (marker effect). All of the previous model terms were considered fixed terms. The random effect ***L*** is associated with the main effect of the genotypes, and **e** corresponds to the error term which captures the unexplained variability.

To control the comparison-wise error rate, the method proposed by Li and Ji (2015) was implemented. The effective number of independent tests (M_eff_) was derived by considering the eigenvalue decomposition of the matrix of correlations between markers. The resulting test was adjusted using the M_eff_ with the following correction (Sidak, 1967):


, $$\alpha_{\text{p}}=1-{\left(1-\alpha_{e}\right)}^{-M_{eff}}$$

Where *α*_p_ is the comparison-wise error rate and *α*_*e*_ corresponds to the experiment error-wise (*α*_*e*_=0.05) .

## Results

### Phenotypic distribution

**Figure 1:**
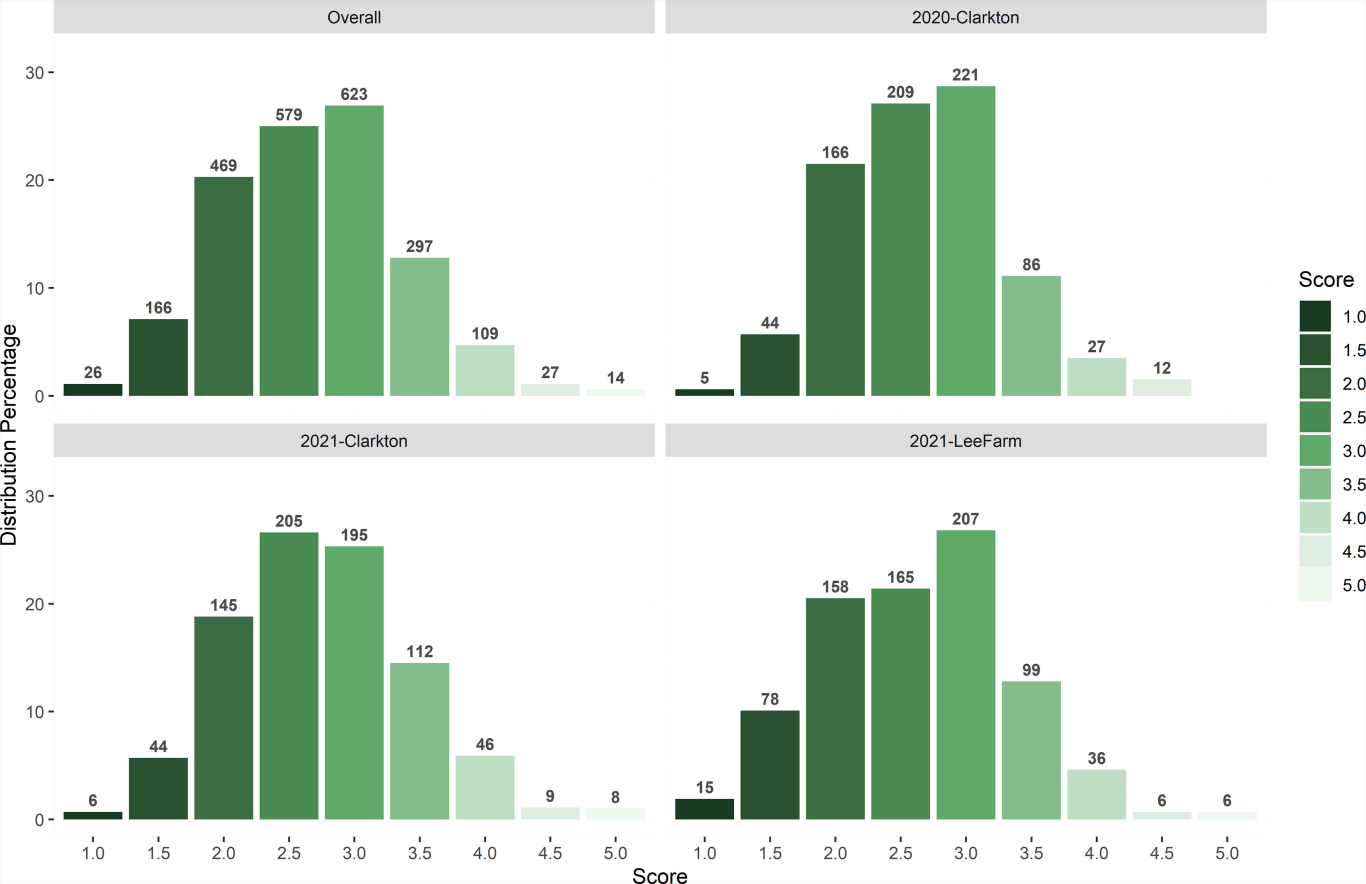
Distribution of off-target dicamba damage scores at each testing environment and across environments.

Across the three testing environments, the frequency of off-target dicamba damage scores was consistent and normally distributed with over 45% of the observations between scores of 2 and 3 (moderately tolerant) and 39% between 3 and 3.5 (moderately susceptible) (**Figure 1**). Roughly 8% of the observations were either under the score of 2 (highly tolerant) or above the score of 4 (highly susceptible).

Across environments, scores were consistent with an overall Cronbach’s alpha (α) score of 0.89 (C.I 0.87 to 0.91). Within environments, scores ranged from 0.85 (2021-Clarkton) to 0.89 (2021-LeeFarm), indicating minor error variance and discrepancy across replications (**Table 1**). Cronbach’s α can be interpreted as the correlation of the test with itself, of which the error variance can be obtained by subtracting the squared α from 1.00 (Tavakol and Dennick, 2011). The error variance ranged from 0.21 to 0.26 which indicates consistency and inter-relatedness of the damage scores across and within environments. Scores above 0.70 (error variance of 0.51) are often considered acceptable (Bland and Altman, 1997; Bosma et al., 1997; McKinley et al., 1997; Kline, 1999; Amirrudin et al., 2020; Canella Vieira et al., 2022b). In plant breeding, reliability refers to multiple measurements across different environments that are independent of each other and may be explored as a new measurement of the influence of genetic versus nongenetic effects as opposed to heritability (Bernardo, 2020).

**Table 1:** Summary of Cronbach’s alpha (α) across testing environments.

Environment	Alpha (α)^1^	C.I (95%)^2^		Error^3^	Correlation^4^	Mean^5^	S.D^6^
Overall	0.89	0.87	0.91	0.21	0.62	2.70	0.59
2020-Clarkton	0.86	0.84	0.88	0.26	0.61	2.70	0.66
2021-Clarkton	0.86	0.85	0.87	0.26	0.61	2.80	0.72
2021-LeeFarm	0.89	0.87	0.91	0.21	0.64	2.60	0.69

^1^Standardized alpha (α) based upon the correlations. ^2^Confidence interval (95%) of standardized alpha (α) score. ^3^Estimated error variance was obtained by subtracting the squared α from 1.00. ^4^Inter-item average Pearson’s correlation. ^5^Average of off-target dicamba damage scores in each environment. ^6^Standard deviation of the observed scores in each year.

### Significant marker-trait associations

**Figure 2:**
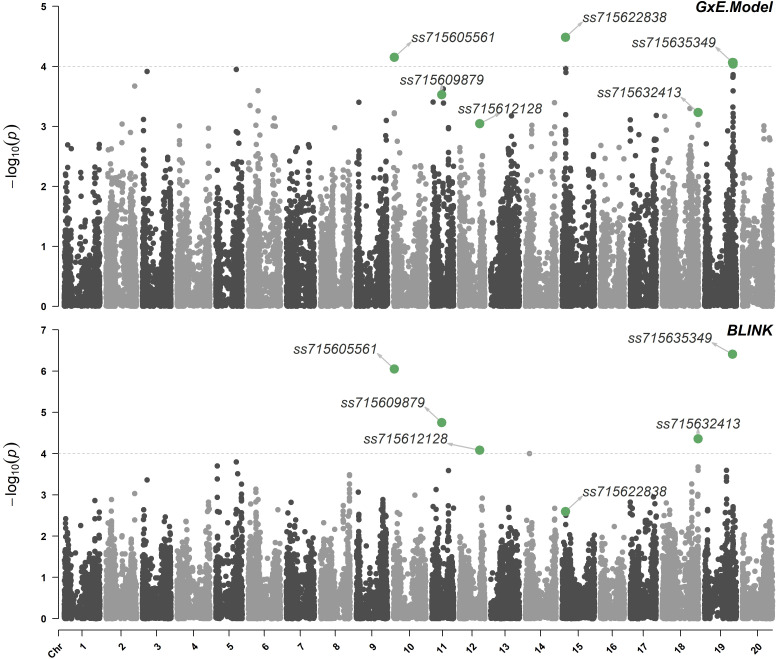
Manhattan plot highlighting significant marker-trait associations using the model that allows the inclusion of the population structure in interaction with environments (G×E) and BLINK. Threshold of marker-trait association significance of approximately 4.0.

The calculated effective number of independent tests (M_eff_) was 575, which returned a threshold of marker-trait association significance of logarithm of odds (LOD) of approximately 4.0. Using the proposed model that allows the inclusion of the population structure in interaction with environments (Eq. 2), three significant marker-trait associations were detected in chromosomes 10 (LG O), 15 (LG E), and 19 (LG L) (**Figure 2**). The SNP *ss715622838* located at 5,457,236 bp of chromosome 15 (Genome assembly version Wm82.a2) had the highest LOD (4.5) with a favorable allele frequency of 13.9%. This SNP is located within the gene *Glyma15g07710* which encodes a copper-containing oxidoreductase enzyme, tyrosinase. These copper containing enzymes can oxidize a wide range of aromatic compounds, including the oxidation of o-diphenols to their corresponding o-quinones (Bijelic et al., 2015; Sullivan, 2015; Min et al., 2019). Phase I of herbicide detoxification in plants involves oxidation by cytochrome P450s or hydrolysis by carboxylesterases (Kreuz et al., 1996; Barrett, 2000). Given the structural similarities between dicamba and o-diphenols, tyrosinase may also be involved in the hydroxylation of dicamba in soybean. The SNP *ss715605561* located at 1,227,933 bp of chromosome 10 (Genome assembly version Wm82.a2) had the second-highest LOD (4.2) with a favorable allele frequency of 8.0%. It is located within the gene *Glyma10g01700* which encodes a multidrug resistance protein (MRP). MRPs are essential in phase III of plant herbicide detoxification by facilitating the transport of glucose- or glutathione-herbicide conjugates into the vacuole (Riechers et al., 2010). Lastly, the SNP *ss715635349* located at 44,580,800 bp of chromosome 19 (Genome assembly version Wm82.a2) had a LOD of 4.1 with a favorable allele frequency of 28.3% (**Table 2**). Interestingly, *ss715635349* is located within the gene *Glyma19g37108*, a uridine diphosphate (UDP)-dependent glycosyltransferase gene. This genomic region contains additional five UDP-glycosyltransferase genes. The conjugation of Phase I-hydroxylated herbicides to endogenous sugar molecules such as glucose is catalyzed by UDP-dependent glycosyltransferases and represents an important phase II reaction of plant herbicide detoxification (Riechers et al., 2010).

**Table 2:** Summary of significant marker-trait associations identified using the G×E model and BLINK.

SNP	Chromosome	Position (bp)^1^	MAF (%)^2^	LOD^3^	
				G×E Model	BLINK
*ss715635349*	19 (LG L)	44,580,800	28.3	4.1	6.4
*ss715605561*	10 (LG O)	1,227,933	8.0	4.2	6.1
*ss715609879*	11 (LG B1)	15,740,804	24.0	3.5	4.8
*ss715632413*	18 (LG G)	57,025,570	19.4	3.2	4.4
*ss715622838*	15 (LG E)	5,457,236	13.9	4.5	2.6
*ss715619759*	14 (LG B2)	6,460,927	19.9	1.7	4.0
*ss715590768*	5 (LG A1)	32,594,828	9.0	3.9	3.8
*ss715592527*	5 (LG A1)	2,516,484	3.3	2.3	3.7
*ss715632432*	18 (LG G)	57,206,151	43.6	3.0	3.7
*ss715632412*	18 (LG G)	57,013,050	26.7	3.0	3.6

^1^Position in the genome reported as base pairs (Genome assembly version Wm82.a2). ^2^Minor allele frequency reported in percentage. ^3^LOD, the logarithm of odds calculated as the negative logarithm of the observed p-value for each model. The G×E model is described in Eq. 2 and BLINK is described in Huang et al. (2019).

Four significant marker-trait associations were detected in chromosomes 10 (LG O), 11 (LG B1), 18 (LG G), and 19 (LG L) using the BLINK model (**Figure 2**). The SNP *ss715635349* located at 44,580,800 bp of chromosome 19 had the highest LOD (6.4) followed by the SNP *ss715605561* located at 1,227,933 bp of chromosome 10 (LOD of 6.1). The SNP *ss715609879* had a LOD of 4.8 with a favorable allele frequency of 24.0%. It is positioned at 15,740,804 bp of chromosome 11 (Genome assembly version Wm82.a2) and is located within *Glyma11g29391*, a lipid phosphate phosphatase gene. Lastly, the SNP *ss715632413* located at 57,025,570 bp of chromosome 18 (Genome assembly version Wm82.a2) had a LOD of 4.4 with a favorable allele frequency of 19.4% (**Table 2**). Two genes with detoxification-related annotations (*Glyma18g291800* and *Glyma18g291700*) are located within 50-kb of *ss715632413* (Genome Browser, www.soybase.org).

### Marker effect on observed phenotype

**Figure 3:**
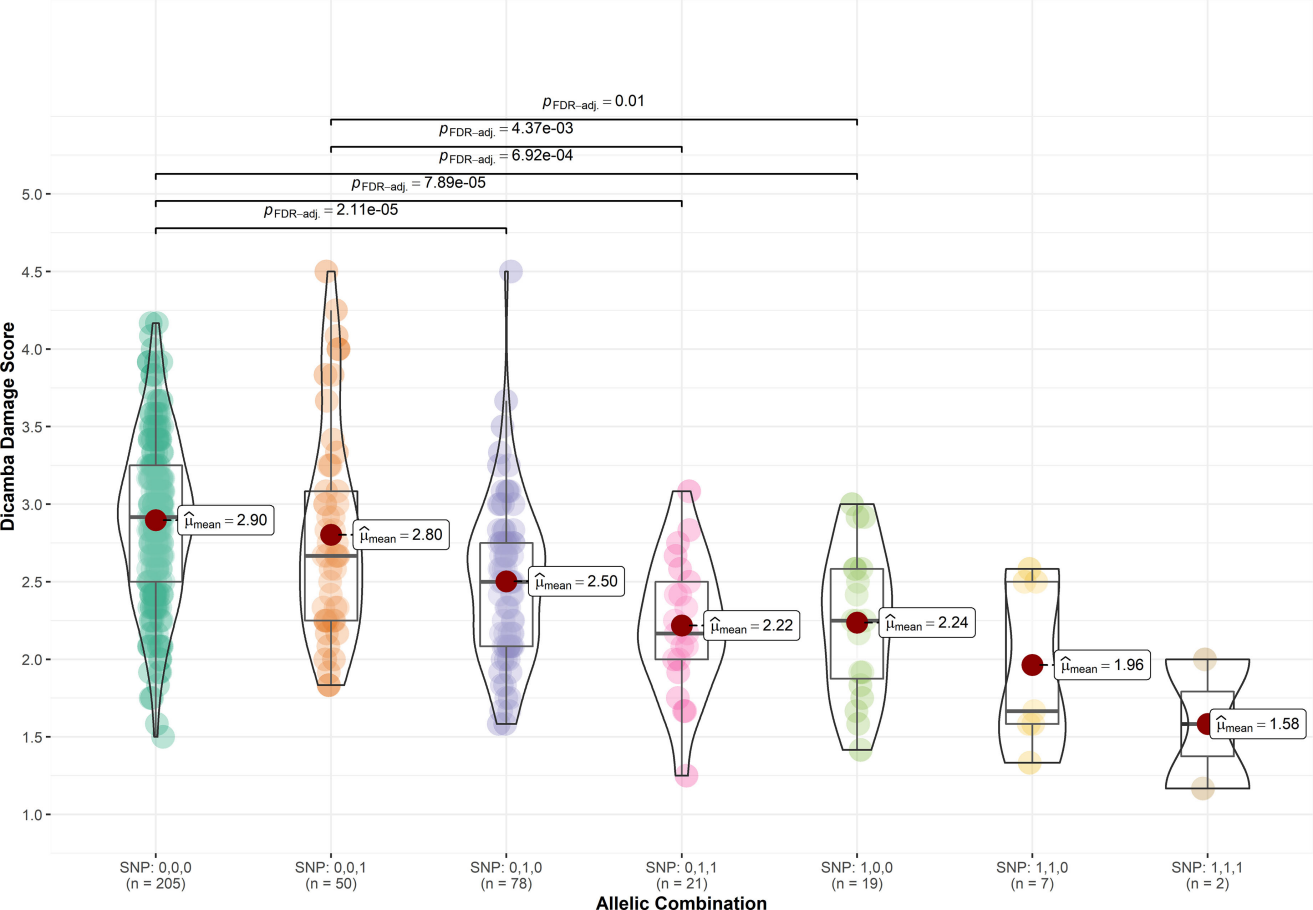
Distribution of off-target dicamba damage scores based on the allelic combination of SNPs *ss715605561* (Chr. 10), *ss715635349* (Chr. 19), and *ss715632413* (Chr. 18). Favorable alleles were represented as 1 whereas unfavorable alleles were represented as 0.

To assess the effect of significant SNPs on the observed phenotype, genotypes were classified according to the allelic combination of the significant SNPs *ss715605561* (Chr. 10), *ss715635349* (Chr. 19), and *ss715632413* (Chr. 18). Favorable alleles were represented as 1 whereas unfavorable alleles were represented as 0. For instance, SNP: 0,0,0 represents the allelic combination where *ss715605561*, *ss715635349*, and *ss715632413* are unfavorable, and SNP: 1,1,1 represents all favorable alleles. The mean score of dicamba damage in genotypes carrying all three favorable alleles was 1.58, whereas the mean score of genotypes carrying all three unfavorable alleles was 2.90 (**Figure 3**). The presence of the favorable allele of *ss715605561* (SNP: 1,0,0; SNP: 1,1,0, and SNP: 1,1,1) and *ss715635349* (SNP: 0,1,0; SNP: 0,1,1; SNP: 1,1,1) significantly reduced the overall damage from off-target dicamba as compared to all non-favorable alleles, while genotypes carrying only the favorable allele for *ss715632413* (SNP: 0,0,1) did not show significant differences to the genotypes carrying only unfavorable alleles (**Figure 3**).

**Figure 4:**
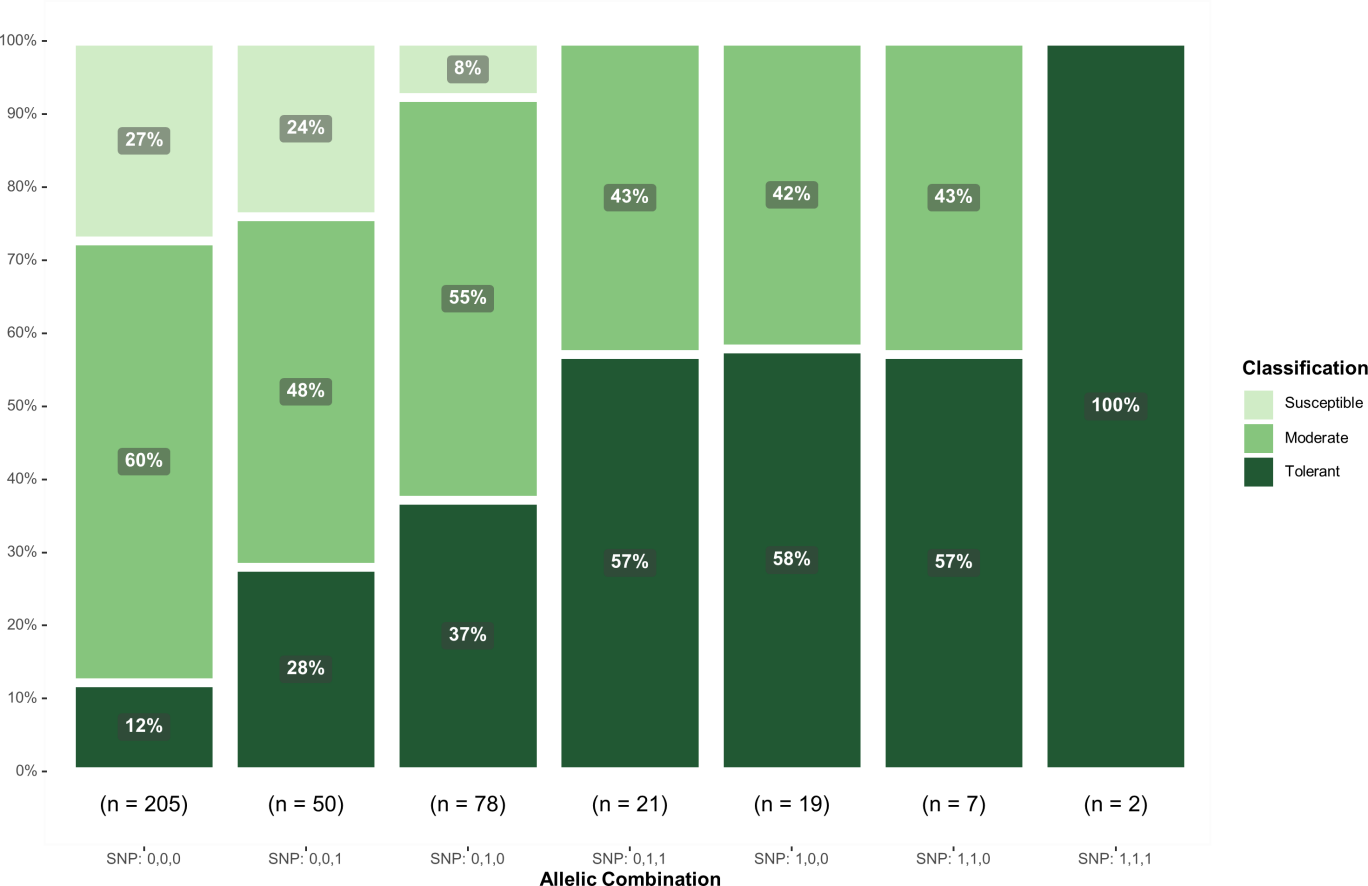
Classification of genotypes based on the allelic combination of SNPs *ss715605561* (Chr. 10), *ss715635349* (Chr. 19), and *ss715632413* (Chr. 18). Favorable alleles were represented as 1 whereas unfavorable alleles were represented as 0.

In addition, to assess the potential of differentiating response classes using the significant SNPs, genotypes were classified as tolerant (score<= 2.5), moderate (2.5< score =< 3.5), and susceptible (score > 3.5). The classification distribution based on the allelic combination of these SNPs showed a substantial reduction in the susceptible class with the inclusion of the favorable alleles of *ss715605561* and *ss715635349* (**Figure 4**). On the other hand, the combination SNP: 0,0,0 had the highest concentration of susceptible (27%) and moderate (60%) genotypes and the lowest concentration of tolerant genotypes (12%) (**Figure 4**). Interestingly, no susceptible genotypes were observed in combinations SNP: 0,1,1, SNP: 1,0,0, SNP: 1,1,0, and SNP: 1,1,1, indicating that the selected SNPs can accurately select genotypes with higher tolerance response to off-target dicamba exposure.

## Discussion

Soybean tolerant to postemergence applications of dicamba was developed under the premise of overcoming weeds resistant to glyphosate as well as allowing rotation and/or mixtures of herbicides to preserve biotechnology-based weed management strategies and maximize its efficacy (Behrens et al., 2007). The insertion of the bacterial gene *dicamba monooxygenase* (*DMO*) from *Pseudomonas maltophilia* (Strain DI-6) encoding the enzyme dicamba *O*-demethylase allows DT plants to metabolize dicamba to 3,6-dichlorosalicylic acid (DCSA), inactivating its herbicidal activity before it accumulates to toxic levels when expressed from either the nuclear genome or chloroplast genome of genetically engineered plants (Herman et al., 2005; Behrens et al., 2007; Wang et al., 2016a). In 2016, the first commercial dicamba-tolerant soybean cultivar was released in the United States and rapidly took over nearly 55% of the soybean acreage. As the incidents of off-target damage widely spread across soybean-growing states, many reports in the literature investigated the relationship between damage and potential yield losses. Soybean is two to six times more sensitive to dicamba when exposed at the early reproductive stage as compared to the vegetative stage (Kelley et al., 2005; Griffin et al., 2013; Robinson et al., 2013; Egan et al., 2014; Solomon and Bradley, 2014; Soltani et al., 2016; Kniss, 2018). Canella Vieira et al. (2022b) estimated yield losses caused by prolonged off-target dicamba exposure in 553 soybean breeding lines derived from 239 unique bi-parental populations. The study reported yield losses of 8.8% for every increment in damage score on a 1–4 scale with losses as high as 40%. Interestingly, certain genetic backgrounds consistently showed natural tolerance to off-target dicamba exposure with minimal symptoms and yield losses.

In this research, a total of 382 genetically diverse soybean accessions ranging from MG 3 to 5 were phenotypically screened based on the severity of damage across three environments subjected to prolonged off-target dicamba exposure. Although each field showed homogenous off-target dicamba distribution, it is practically impossible to assess the total dosage of dicamba received by each experimental plot in specific growth stages and throughout the season. Therefore, controlled dose-response experiments in the absence of off-target dicamba exposure should be conducted to precisely identify the threshold of which the identified genomic regions can sustain tolerance responses. Most accessions showed a moderate response, either moderately tolerant or moderately susceptible, with approximately 8% showing high tolerance (scores< 2) and susceptibility (scores > 4). No differences in off-target dicamba damage were observed across MG (**Supplementary Table 1**, MG 3: average damage of 2.8, MG 4: average damage of 2.7, MG 5: average damage of 2.5). Late-maturing soybean genotypes are associated with lower off-target dicamba damage due to a longer window to detoxify from low rates of dicamba between planting and flowering compared with early-maturing genotypes (Wax et al., 1969; Weidenhamer et al., 1989; McCown et al., 2018). Tolerant soybean accessions were identified across all MG, confirming that natural tolerance to off-target dicamba may be caused by physiological mechanisms other than the length of time for recovery (Canella Vieira et al., 2022b). In addition, no substantial geographical effects have been identified across continents, although accessions derived from Asia (average damage of 2.6) had on average lower off-target dicamba damage as compared to accessions derived from the Americas (average damage of 3.0). Specifically, South Korea (average damage of 2.5), Japan (average damage of 2.6), and China (average damage of 2.7) had on average lower off-target dicamba damage as compared to accessions derived from the United States (average damage of 2.3) and Costa Rica (average damage of 3.1), although the number of accessions was highly unbalanced across countries (**Supplementary Table 1**).

Plant introduction (PI) 424005 (average damage of 1.2, South Korea), PI 424038-B (average damage of 1.3, South Korea), PI 561701 (G88-20092, average damage of 1.4, United States), PI 603497 (average damage of 1.4, China), and PI 342434 (average damage of 1.5, Japan) were the five most tolerant accessions (**Supplementary Table 1**). On the other hand, PI 547862 (L83-570, average damage of 4.1, United States), PI 552538 (Dunbar, average damage of 4.2, United States), PI 598124 (Maverick, average damage of 4.3, United States), PI 603675 (average damage of 4.5, China), and PI 597387 (Pana, average damage of 4.5, United States) were the five most susceptible (**Supplementary Table 1**). Interestingly, four out of the five most susceptible accessions are genetically related to cultivars that widely contributed to the genetic basis of modern soybean cultivars in the United States (Gizlice et al., 1993; Gizlice et al., 1994; Carter et al., 2004; Hyten et al., 2006; Vieira and Chen, 2021). For instance, Maverick and Pana are derived from LN86-4668, which is a progeny of Fayette (PI 518674, direct progeny of PI 88788). PI 88788, which has been widely used as a genetic source of resistance to soybean cyst nematode (*Heterodera glycine Ichinohe*), is susceptible to off-target dicamba (average damage of 3.5). In general, soybean [*Glycine max* (L.) Merr.] shows a moderate response to off-target dicamba, and yield losses are expected when prolonged exposure occurs (Canella Vieira et al., 2022b). Furthermore, genetic variation conferring higher tolerance to off-target dicamba appears to be rare in landraces, highlighting the value of the USDA Soybean Germplasm Collection to restore economic-important alleles lost during domestication and intensive breeding (Gizlice et al., 1993; Gizlice et al., 1994; Carter et al., 2004; Hyten et al., 2006; Vieira and Chen, 2021). This has been the case in multiple economically important traits, including resistance to soybean cyst nematode (Anand et al., 1988; Young, 1990), root-knot nematodes (*Meloidogyne* spp.) (Luzzi et al., 1987), foliar feeding insects (van Duyn et al., 1971), and brow stem rot (*Phialophora gregata*) (Chamberlain and Bernard, 1968).

Two models were used to identify marker-trait associations regulating the response of soybean to off-target dicamba. BLINK minimizes false-positive associations and greatly improve computational efficiency in larger datasets (Huang et al., 2019). To account for possible underlying population structures affecting the observed response to off-target dicamba in different environments, a model including the first 10 principal components (PC) derived from decomposing the G matrix as well as the interaction between each PC and the environment was developed. In addition, this model allows the inclusion of all observed phenotypes (three environments × two replications per genotype) as opposed to only one adjusted mean per genotype, substantially reducing the ‘Curse of Dimensionality’ where the number of independent variables is far higher than the number of samples that are often seen in genomic studies (Nicholls et al., 2020). We observed that both models identified significant associations between *ss715635349* (Chr. 19) and *ss715605561* (Chr. 10) and the response to off-target dicamba. The BLINK model identified additional significant marker-trait associations on Chrs. 11 (*ss715609879*), 14 (*ss715619759*), and 18 (*ss715632413*), while the G×E Model identified an additional significant marker-trait association on Chr. 15 (*ss715622838*). The significant SNPs identified by both models are located within candidate genes with annotated functions involved with different phases of herbicide detoxification in herbicides.

Phase I typically involves oxidation by cytochrome P450s or hydrolysis by carboxylesterases. These reactions introduce a reactive functional group suitable for subsequent metabolism and detoxification since this initial oxidation step may not lead to complete detoxification (Kreuz et al., 1996; Barrett, 2000). Phase II detoxification reactions involve the conjugation of herbicides with reduced glutathione or glucose and are catalyzed by glutathione S-transferases or UDP-dependent glycosyltransferases (Riechers et al., 2010). The SNP *ss715635349* is located within the gene *Glyma19g37108*, a uridine diphosphate (UDP)-dependent glycosyltransferase gene. Within a 30-kb window from *ss715635349* (44,550,000 to 44,610,000 bp) there are another five UDP-dependent glycosyltransferases genes (Genome Browser, www.soybase.org ). Given the frequency of the favorable allele of *ss715635349*, we hypothesize that the ability to complete phase II detoxification of dicamba is relatively common (28.3%) and may explain the overall moderate response of soybean to off-target dicamba. Phase III of herbicide detoxification involves the active transport (ATP-dependent) of non-phytotoxic herbicide conjugates into the vacuole by proteins in the multidrug resistance-associated protein (MRP) family or by other transport mechanisms (Kreuz et al., 1996; Riechers et al., 2010). The SNP *ss715605561* is located within the MRP gene *Glyma10g01700*. The low favorable allele frequency (8.0%) could explain the rare occurrence of highly dicamba-tolerant soybean phenotypes. Based on these results, we hypothesize that most soybean genotypes conduct phase I ring hydroxylation and phase II detoxification of dicamba with glucose but have a rate-limiting step in the final phase III transport of non-phytotoxic dicamba-glucose conjugates into the vacuole. However, further metabolite profiling and subcellular transport studies are warranted to test this hypothesis directly.

Although the response to off-target dicamba appears to be a highly complex trait regulated by multiple genes involved in several biochemical pathways, the combination of *ss715605561* (Chr. 10), *ss715635349* (Chr. 19), and *ss715632413* (Chr. 18) can accurately distinguish among tolerant to susceptible genotypes. Accessions carrying the favorable alleles for these SNPs showed the lowest average off-target dicamba damage and the highest frequency of tolerant and moderate classes. Future plant breeding research will utilize and apply these alleles in marker-assisted selection programs targeting identification and development of genotypes with higher tolerance to off-target dicamba. Additionally, molecular physiology research is currently underway to investigate expression patterns and functional roles of the alleles and encoded proteins identified by our GWAS analysis.

## Conclusions

Widespread adoption of DT production systems has frequently resulted in yield losses in non-DT soybean genotypes from off-target dicamba exposure. Identification of genetic sources of tolerance and genomic regions conferring higher tolerance to off-target dicamba in non-DT soybean genotypes may sustain and improve other non-DT soybean production systems, including the growing niche markets of organic and conventional soybean. Herein, we report several genetically diverse accessions that can be used as genetic sources for improved tolerance to off-target dicamba. Two genomic regions on Chrs. 10 and 19 were identified that may be directly associated with the ability of soybean to detoxify dicamba and/or transport non-phytotoxic dicamba metabolites into the vacuole. Three significant SNPs accurately distinguished between tolerant and susceptible genotypes. Intensive breeding that targeted single economically important traits may have caused selective sweeps where alleles conferring tolerance to off-target dicamba were lost after many breeding cycles. With the advancements in targeted gene-editing techniques, our results may facilitate identifying and developing conventional soybean cultivars with improved tolerance to off-target dicamba as well as other synthetic auxin herbicides. Further research investigating the physiological mechanisms underlying natural tolerance to dicamba in soybean and dose-response studies in controlled environments can help to determine the threshold at which tolerant genotypes maintain minimal symptomology following off-target dicamba exposure.

## Data availability statement

The datasets presented in this study can be found in online repositories. The name of the repository and link to the data can be found as follows: Dryad; https://doi.org/10.5061/dryad.gmsbcc2s4.

## Author contributions

CC, DJ, JFZ, BD, DR, DX, GS, PC, and HN contributed to conception and design of the study. CC, BD, DR, and PC contributed to funding resources of the study. CC, EdN, DL, and SS contributed to collection of data used in this study. CC, DJ, and JZ contributed to the statistical analysis of this study. CC wrote the first draft of the manuscript. All authors contributed to manuscript revision, read, and approved the submitted version.
